# 

**Published:** 1860-11

**Authors:** 


CONTENTS

OF THE

### Chicago Medical Journal for November, 1860.

### ORIGINAL COMMUNICATIONS.

ARTICLE 1—Case of Spina Bifida. By John Low, M. D. .................................. 633
ARTICLE 2—Case of Suicide by Opium and Strychnine. Reported by J. S. Pashley, M. D... 635
ARTICLE 3—Cancrum Oris. By Charles Brackett, M. D. .................................. 638
ARTICLE 4—Inflammation of the Parotid Glands. By Charles Brackett, M. D. ........... 640

### EDITORIAL.

Book and Pamphlet Notices.......................................... 641 to 661, and 673 to 680

### SELECTED.

Extracts from an Essay on Diphtheria and Diphtheritic Affections ....................... 661
Medical Education—Its Tendencies ................................................ 681
Practical Clinical Remarks—On Tetanus ........................................... 684
Treatment of Lupus ............................................................... 689
Lecture on the Local Effects of General Diseases .................................. 691

